# Gender-specific association between serum ferritin and neurodevelopment in infants aged 6 to 12 months

**DOI:** 10.1038/s41598-023-29690-x

**Published:** 2023-02-13

**Authors:** Yong Guo, Li Yu, Zi-Yu Wu, Yu-Hong Deng, Jie-Ling Wu

**Affiliations:** 1 Department of Children’s Health Care, Guangdong Women and Children Hospital, Guangzhou Medical University, Guangzhou, 511400 China; 2 Department of Medical Ultrasound, Guangdong Women and Children Hospital, Guangzhou Medical University, Guangzhou, 511400 China

**Keywords:** Health care, Nutrition, Paediatrics

## Abstract

Early iron deficiency has detrimental consequences on neurodevelopment; whether male and female infants are equally susceptible to the functional outcomes of iron deficiency is unclear. This study aimed to investigate the sex differences in the association between serum ferritin levels and neurodevelopment in infants. Data for this cross-sectional study were drawn from hospital information and early childhood development program service systems at Guangdong Women and Children’s Hospital, Guangzhou, China. In total, 4579 infants aged 6–12 months were included from July 2018 to March 2020. Their neurodevelopment was assessed using the Children Neuropsychological and Behavior Scale-Revision 2016. Serum ferritin levels were measured by chemiluminescence assay. The association between serum ferritin levels and neurodevelopmental delay in each domain was estimated using logistic regression models adjusted for potential confounders. The mean concentration of serum ferritin was 35.56 ± 21.57 ng/mL. Serum ferritin levels were significantly higher in female than in male infants (*P* < 0.001). Iron deficiency (serum ferritin levels < 12 ng/mL) was significantly more prevalent in male than in female infants (*P* < 0.001). Linear regression revealed a positive association between serum ferritin levels and general quotient, gross motor, fine motor, language, and adaptive behavior in females. Iron deficiency was significantly associated with an increased risk of adaptive behavior delay in females (adjusted odds ratio (OR), 2.22; 95% confidence interval (CI): 1.17–4.20). Iron deficiency anemia was associated with an increased risk of developmental delay for general quotient (adjusted OR, 4.88; 95% CI: 1.74–13.65), fine motor (adjusted OR = 2.58, 95%: CI: 1.13–5.94) and adaptive behavior (adjusted OR, 3.38; 95% CI: 1.51–7.57) among females, but not in males. Associations between serum ferritin levels and neurodevelopment in infants aged 6–12 months were sex-related. Females with iron deficiency, especially those with iron-deficiency anemia, were more susceptible to neurodevelopmental delay than males.

## Introduction

Serum ferritin is a reliable indicator of body iron stores and is commonly used to diagnose and monitor iron deficiency^1^. Serum ferritin levels decrease because of iron deficiency and anemia. Iron deficiency is a prevalent nutritional deficiency in early childhood^2^ that could have a negative impact on neurodevelopment and has been linked to long-term neurobehavioral consequences, including poor attention, increased anxiety, and depression^3–6^. Although efforts have been made to establish links between serum ferritin levels and neurodevelopmental function in humans, few studies have explored sex-specific relationships between serum ferritin concentrations and neurodevelopment. It is generally believed that the differences in iron status between males and females arise after adolescence^7,8^. Some studies have reported substantial sex differences in serum ferritin levels in infants and pre-pubertal children^9–11^. Serum ferritin levels differ significantly according to sex, suggesting a sex-dependent relationship for ferritin and neurodevelopmental function risk. We therefore set out to investigate the sex differences in the association between serum ferritin and neurodevelopment in infants aged 6–12 months.

## Methods

### Study design and participants

The sample for this cross-sectional study was drawn from database of the hospital information system and early childhood development program service system. The early childhood development program service system was used to monitor the growth of children with regular health checkups at the Guangdong Women and Children’s Hospital, Guangzhou, China. When children aged < 6 years underwent routine health check-ups, all data on maternal information, physical measurements, and neurodevelopmental examinations were recorded. In the present analysis, we included 6–12-month-old infants who underwent routine health checkups. Data on neurodevelopmental measurements were extracted from the early childhood development program service system from July 2018 to March 2020. These data were linked to individual serum ferritin levels and hemoglobin records from the hospital information system. Regarding potential confounding factors, the choice of covariates that may have confounded the relationship between serum ferritin levels and childhood neurodevelopment was guided by directed acyclic graphs (Supplementary Fig. S1). We identified several variables obtained from medical records and the early childhood development program service system. Factors extracted included maternal education, parity, feeding at 6 months, infant age, height, and weight. Infants were excluded if they had premature birth, or were diagnosed with a hematologic disorder such as thalassemia, congenital diseases, gastrointestinal disorders, chronic inflammation, or infection. Based on the inclusion and exclusion criteria, 4579 infants aged 6–12 months were enrolled in the final analysis.

### Ethical statement

This study was approved by the Medical Research Ethics Board of Guangdong Women and Children’s Hospital. As this was a retrospective study utilizing data from the existing hospital laboratory information system, the Ethics Committee of Guangdong Women and Children’s Hospital waived the need for informed consent. The accessed patient data complied with relevant data protection and privacy regulations.

### Laboratory assessments

Ferritin and hemoglobin measurements were extracted from the hospital’s laboratory information system. During our study period, to minimize the impact of potential batch effect on laboratory measurements, the ferritin and hemoglobin laboratory tests were performed according to the consistent platform and standard operating procedures. Serum ferritin was measured by a chemiluminescence assay using an Abbott i2000SR analyzer^12^, and hemoglobin was measured using an automated hematology analyzer (Siemens Advia 2120i) as described elsewhere^13^. Based on the World Health Organization criteria for using ferritin concentrations to assess iron status in infants and young children aged 0–23 months^14^, iron deficiency was defined as a serum ferritin level < 12 ng/mL and iron deficiency anemia (IDA) as a serum ferritin level < 12 ng/mL and hemoglobin < 110 g/L.

### Neurodevelopmental assessment

Neurodevelopmental levels of the infants were assessed using the Children Neuropsychological and Behavior Scale-Revision 2016 (CNBS-R2016). The CNBS-R2016 is a diagnostic assessment tool developed by the Capital Institute of Pediatrics in China that is widely used to assess the developmental level of children aged 0–6 years^15,16^. It includes general quotient and five subscales: gross motor, fine motor, language, personal-social, and adaptive behaviors. A general or subscale quotient < 80 points indicates a mild delay (< 70 points means a significant delay), and a quotient ≥ 80 points indicates no delay.

### Statistical analysis

Data are presented as the mean ± standard deviation for continuous variables and as numbers (percentages) for categorical variables. For the comparison of differences between male and female infants, the t-test was used for continuous variables and the chi-square test was used for categorical variables. Correlations between serum ferritin levels and the different domain scores of the CNBS-R2016 were analyzed using Pearson’s correlation analysis. The linear association between serum ferritin levels and the different domain scores of the CNBS-R2016 was tested using linear regression models. In the adjusted models, some covariates, including maternal education, parity, feeding at 6 months, age of the infant, height, and weight, were considered as potential confounders as they were reported to be related to neurodevelopment or serum ferritin levels based on previous studies. The odds ratios (ORs) and 95% confidence intervals (CIs) for the association between serum ferritin (< 12 ng/mL vs.  ≥ 12 ng/mL) and neurodevelopmental delay (< 80 points) in each domain were estimated using logistic regression models, considering the serum ferritin level ≥ 12 ng/mL group as the reference category, and adjusting for maternal education, parity, feeding at 6 months, infant age, height, and weight. In addition, we performed analyses for the association between IDA (yes: serum ferritin < 12 ng/mL and hemoglobin < 110 g/L vs. no) and neurodevelopmental delay. We conducted separate experiments in males and females to evaluate whether infant sex modified the relationship between serum ferritin levels and neurodevelopment. Interactions between infant sexes were tested by including an interaction term of infant sex × serum ferritin in the corresponding full model and obtaining a *p*-value for the interaction. R software version 4.1.0 (www.R-project.org) and the SPSS statistical software package (V20, IBM Statistics, Chicago, IL, USA) were used for all statistical analyses. *P* < 0.05 was considered to be the threshold for statistical significance in analyses.

## Results

Characteristics of the infants according to sex are presented in Table 1. Among the 4579 infants, the mean age was 8.47 ± 2.25 months, and 2660 (58.1%) were males. The mean concentration of serum ferritin was 35.56 ± 21.57 ng/mL. Serum ferritin levels were significantly higher in female than in male infants (means 38.31 ng/mL and 33.58 ng/mL, respectively; *P* < 0.001). Iron deficiency (defined as a serum ferritin level < 12 ng/mL) was also significantly more prevalent in male infants (12.6%) than in female infants (7.8%). The total proportion of infants with IDA was 5.8%, with a significant sex difference (*P* < 0.001).

**Table 1:** Characteristics of the infants (n = 4579).

Variables	All (n = 4579)	Male (n = 2660)	Female (n = 1919)	*t*/χ^2^ value	*P* value
Maternal education					
Junior high school or below	261 (5.7)	177 (6.7)	84 (4.4)	41.764	< 0.001
Senior high school	806 (17.6)	441 (16.6)	365 (19.0)		
College	1238 (27.0)	743 (27.9)	495 (25.8)		
Undergraduate or above	2016 (44.0)	1189 (44.7)	827 (43.1)		
Missing	258 (5.6)	110 (4.1)	148 (7.7)		
Parity, n (%)					
1	2737 (59.8)	1578 (59.3)	1159 (60.4)	0.534	0.465
≥ 2	1842 (40.2)	1082 (40.7)	760 (39.6)		
Feeding at six months, n(%)					
Breastfed exclusively	1512 (33.0)	829 (31.2)	683 (35.6)	9.890	0.007
Mixed	2133 (46.6)	1275 (47.9)	858 (44.7)		
Formula-fed exclusively	934 (20.4)	556 (20.9)	378 (19.7)		
Infant age (months)	8.47 ± 2.25	8.39 ± 2.23	8.59 ± 2.28	2.841	0.005
Height (cm)	70.23 ± 3.66	70.74 ± 3.61	69.53 ± 3.61	11.258	< 0.001
Weight (kg)	8.31 ± 1.10	8.53 ± 1.11	8.01 ± 0.99	16.047	< 0.001
Serum ferritin (ng/mL)	35.56 ± 21.57	33.58 ± 20.84	38.31 ± 22.26	7.355	< 0.001
Serum ferritin < 12 ng/mL, n(%)	485 (10.6)	335 (12.6)	150 (7.8)	26.867	< 0.001
Serum ferritin ≥ 12 ng/mL, n(%)	4094 (89.4)	2325 (87.4)	1769 (92.2)		
Hemoglobin (g/L)	114.58 ± 9.18	114.37 ± 9.54	114.88 ± 8.65	1.806	0.071
Hemoglobin < 90 g/L, n(%)	52 (1.1)	35 (1.3)	17 (0.9)	9.965	0.007
Hemoglobin 90–110 g/L, n(%)	1044 (22.8)	645 (24.2)	399 (20.8)		
Hemoglobin ≥ 110 g/L, n(%)	3483 (76.1)	1980 (74.4)	1503 (78.3)		
IDA, n(%)	264 (5.8)	192 (7.2)	72 (3.8)	24.650	< 0.001

Values are presented as the mean ± SD or n(%). Differences between the male and female groups were explored using the t-test or chi-square test. IDA, iron deficiency anemia.

**Figure 1:**
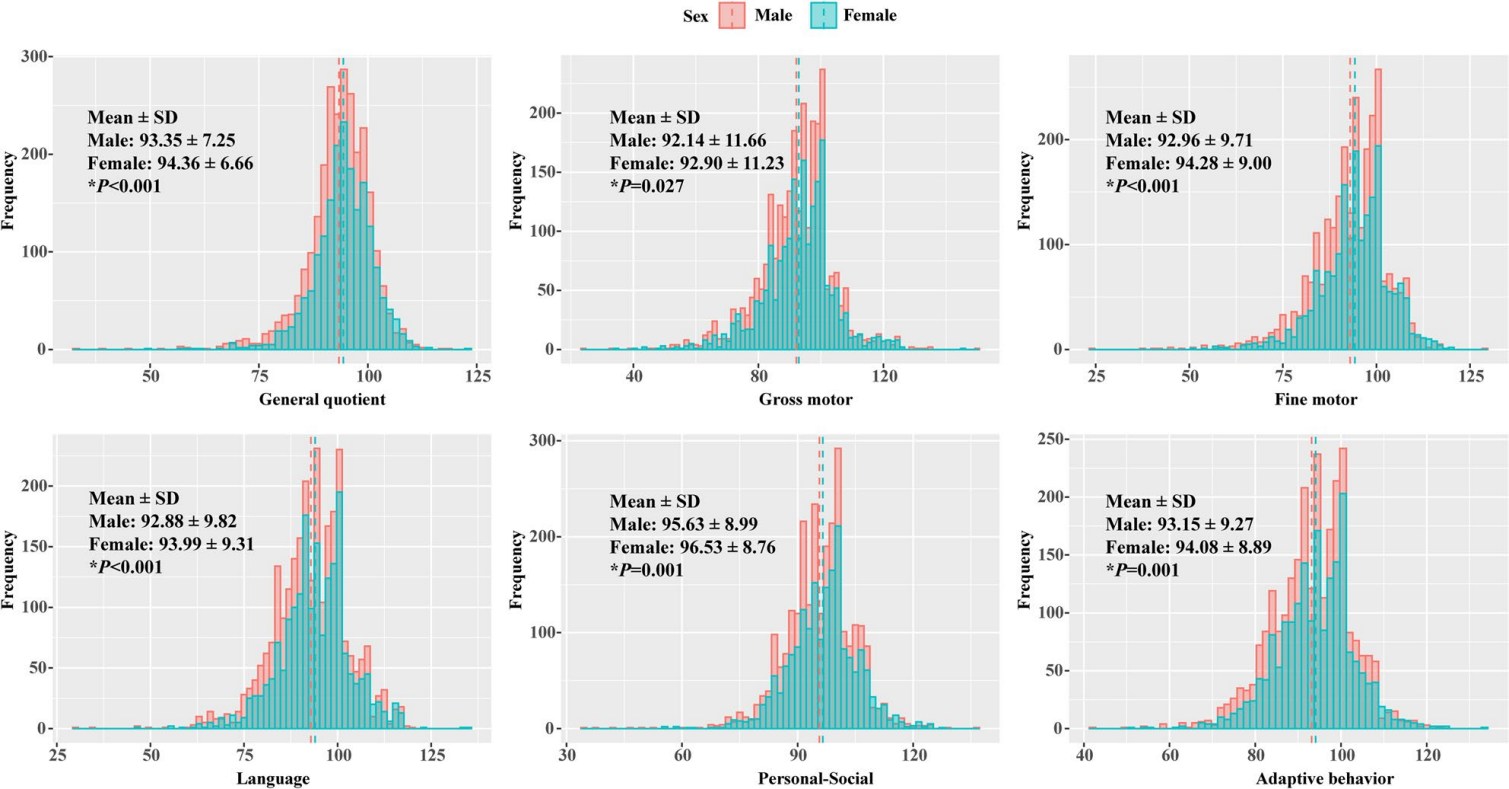
Distribution of CNBS-R2016 scores in different neurodevelopmental domains grouped by sex. Histograms depict the distribution of CNBS-R2016 scores for male (red) and female (blue) infants. The dashed lines in each histogram are the mean values. Differences in scores between males and females were determined by independent samples t-test. CNBS-R2016, Children Neuropsychological and Behavior Scale-Revision 2016.

The CNBS-R2016 scores in different neurodevelopmental domains presented significant sex-specific findings in general quotient, gross motor, fine motor, language, personal-social, and adaptive behaviors (Fig. 1). The mean scores of the general quotients and five subscales were significantly higher in females than in males (all *P* < 0.05). Neurodevelopmental delays in general quotient, gross motor, fine motor, language, and personal-social that occurred in males with iron deficiency were similar to those in females. Adaptive behavior delays occurred more frequently in females with iron deficiency than in males (Table 2).

**Table 2:** Comparison of developmental delays by sex and serum ferritin levels.

Variable	Total	Male ferritin		*P* value	Female ferritin		*P* value
		< 12 ng/mL	≥ 12 ng/mL		< 12 ng/mL	≥ 12 ng/mL	
General quotient							
No delay	4441 (97.0)	326 (97.3)	2236 (96.2)	0.300	145 (96.7)	1734 (98.0)	0.265
Delay	138 (3.0)	9 (2.7)	89 (3.8)		5 (3.3)	35 (2.0)	
Gross motor							
No delay	4111 (89.8)	299 (89.3)	2070 (89.0)	0.903	136 (90.7)	1606 (90.8)	0.961
Delay	468 (10.2)	36 (10.7)	255 (11.0)		14 (9.3)	163 (9.2)	
Fine motor							
No delay	4284 (93.6)	308 (91.9)	2153 (92.6)	0.667	142 (94.7)	1681 (95.0)	0.847
Delay	295 (6.4)	27 (8.1)	172 (7.4)		8 (5.3)	88 (5.0)	
Language							
No delay	4269 (93.2)	312 (93.1)	2148 (92.4)	0.628	141 (94.0)	1668 (94.3)	0.883
Delay	310 (6.8)	23 (6.9)	177 (7.6)		9 (6.0)	101 (5.7)	
Personal-Social							
No delay	4443 (97.0)	325 (97)	2249 (96.7)	0.784	145 (96.7)	1724 (97.5)	0.560
Delay	136 (3.0)	10 (3.0)	76 (3.3)		5 (3.3)	45 (2.5)	
Adaptive behavior							
No delay	4308 (94.1)	315 (94.0)	2168 (93.2)	0.591	137 (91.3)	1688 (95.4)	0.026
Delay	271 (5.9)	20 (6.0)	157 (6.8)		13 (8.7)	81 (4.6)	

Differences in the % developmental delay were compared using the chi-square test.

**Figure 2:**
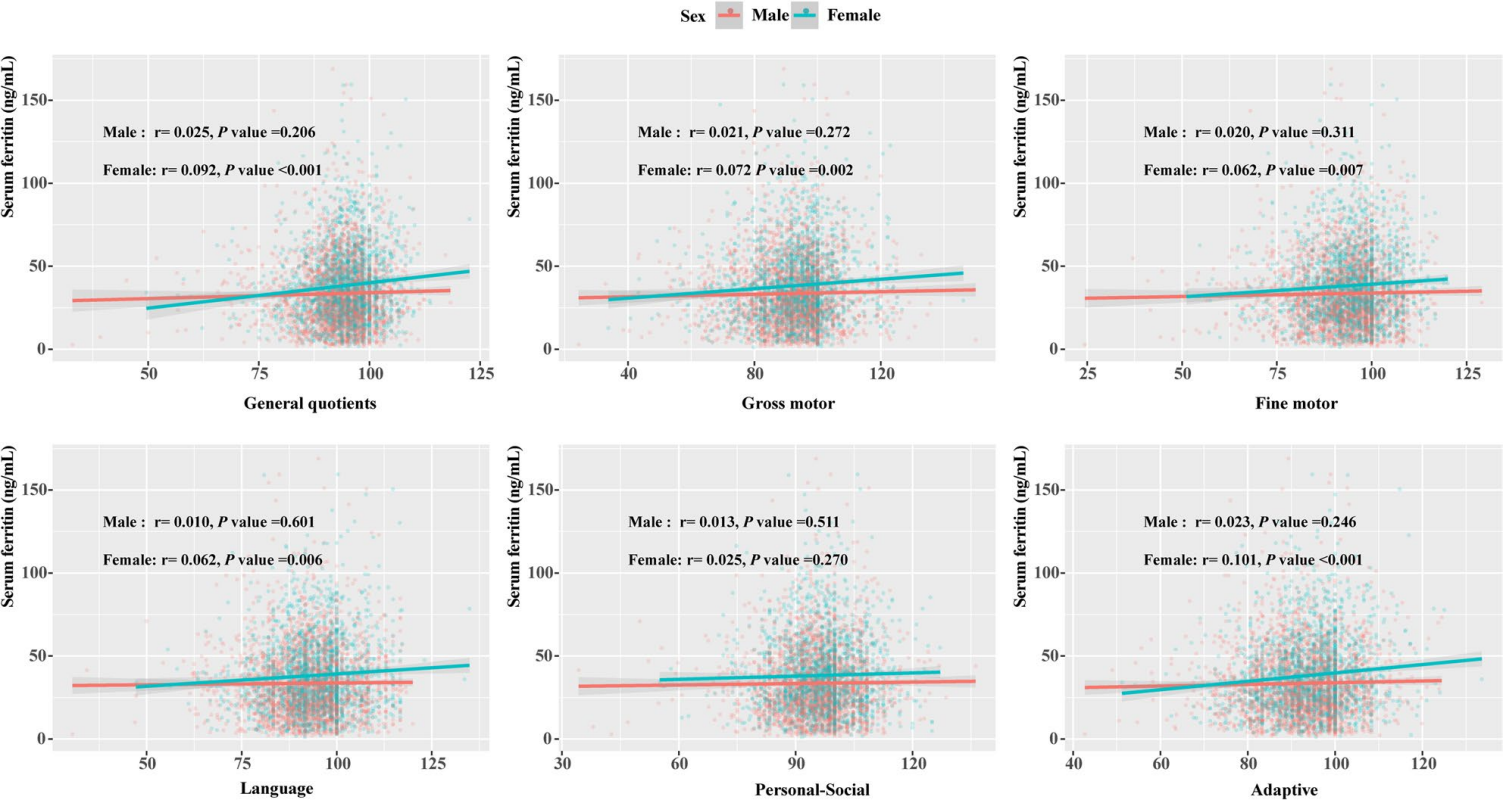
Correlations between CNBS-R2016 scores across domains and serum ferritin levels by sex. CNBS-R2016, Children Neuropsychological and Behavior Scale-Revision 2016.

CNBS-R2016 scores across domains (except for personal-social) were positively correlated with serum ferritin levels in females but not in males (Fig. 2). In the linear regression adjusted model, CNBS-R2016 scores of different neurodevelopmental domains showed similar sex-specific associations with serum ferritin levels (Table 3).

**Table 3:** Association of serum ferritin levels and developmental function.

Variable	Total		Male		Female	
	β (95% CI)	*P* value	β (95% CI)	*P* value	β (95% CI)	*P* value
General quotient	0.016 (0.006, 0.025)	0.001	0.002 (− 0.011, 0.016)	0.721	0.022 (0.009, 0.035)	0.001
Gross motor	0.024 (0.008, 0.039)	0.003	0.008 (− 0.014, 0.029)	0.495	0.035 (0.012, 0.058)	0.002
Fine motor	0.015 (0.002, 0.027)	0.024	0.001 (− 0.017, 0.019)	0.936	0.020 (0.002, 0.038)	0.033
Language	0.014 (0.001, 0.027)	0.040	0.001 (− 0.016, 0.019)	0.944	0.019 (0.001, 0.038)	0.040
Personal-Social	0.005 (− 0.007, 0.017)	0.449	− 0.001 (− 0.017, 0.016)	0.946	0.003 (− 0.014, 0.021)	0.700
Adaptive behavior	0.021 (0.009, 0.033)	0.001	0.003 (− 0.014, 0.020)	0.762	0.034 (0.016, 0.051)	< 0.001

Linear regression models adjusted for maternal education, parity, feeding at six months, infant age, height, and weight.

**Figure 3:**
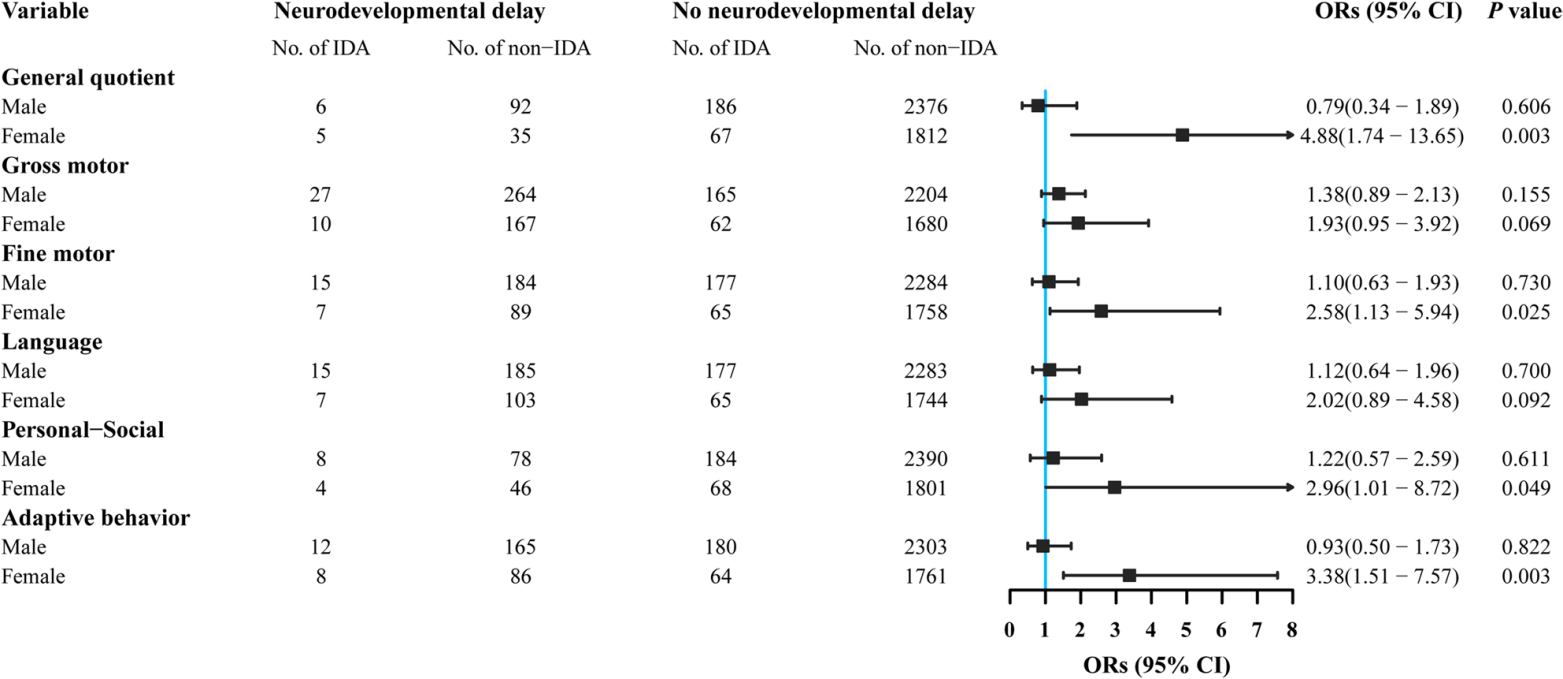
Associations between IDA (serum ferritin < 12 ng/mL and hemoglobin < 110 g/L) and neurodevelopmental delays for males and females. The ORs were adjusted for maternal education, parity, feeding at six months, infant age, height, and weight. IDA, iron deficiency anemia; ORs, odds ratios.

The estimated ORs (95% CIs) of developmental delay for each domain according to sex-specific iron deficiency using logistic regression analysis are presented in Table 4. Iron deficiency was significantly associated with an increased risk of adaptive behavior delay in females, but not in males. In an additional analysis that tested for sex differences in the association between IDA and neurodevelopmental delay, we found that IDA was associated with an increased risk of developmental delay for general quotient (adjusted OR, 4.88; 95% CI: 1.74–13.65), fine motor (adjusted OR, 2.58; 95% CI: 1.13–5.94), and adaptive behavior (adjusted OR, 3.38; 95% CI: 1.51–7.57) among females but not in males (Fig. 3). Furthermore, the interaction effect of sex was significant for the general quotient and adaptive behavior when controlling for potential confounders (*P* = 0.015 and *P* = 0.030, respectively; Table S1).

**Table 4:** Logistic regression analyses of neurodevelopmental delays and iron deficiency (serum ferritin < 12 ng/mL vs. serum ferritin ≥ 12 ng/mL).

Variable	Total		Male		Female		*P*-Interaction
	ORs (95% CI)	*P* value	ORs (95% CI)	*P* value	ORs (95% CI)	*P* value	Sex ∗ serum ferritin
General quotient	0.99 (0.56, 1.77)	0.991	0.66 (0.32, 1.35)	0.256	1.78 (0.66, 4.78)	0.251	0.132
Gross motor	1.10 (0.80, 1.51)	0.549	0.99 (0.68, 1.45)	0.958	1.13 (0.63, 2.03)	0.685	0.923
Fine motor	1.23 (0.85, 1.78)	0.275	1.13 (0.73, 1.74)	0.588	1.17 (0.55, 2.51)	0.676	0.957
Language	1.05 (0.71, 1.54)	0.82	0.92 (0.58, 1.46)	0.742	1.12 (0.55, 2.28)	0.751	0.703
Personal-Social	1.08 (0.62, 1.87)	0.788	0.84 (0.43, 1.67)	0.625	1.48 (0.57, 3.86)	0.415	0.557
Adaptive behavior	1.22 (0.83, 1.80)	0.306	0.84 (0.51, 1.37)	0.477	2.22 (1.17, 4.20)	0.014	0.034

The ORs were adjusted for maternal education, parity, feeding at six months, infant age, height, and weight.

*P* value for the interaction between sex and serum ferritin, with the following variables also included in the model: maternal education, parity, feeding at six months, infant age, height, and weight.

*ORs* Odds ratios, *CI* Confidence interval.

## Discussion

In the present study, we performed a comprehensive analysis of sex-specific associations between serum ferritin levels and neurodevelopmental function, based on data from the hospital’s early childhood development program service system. We observed that serum ferritin concentration varied by sex and was positively associated with developmental scores of general quotient, gross motor, fine motor, language, and adaptive behavior in female infants but not in male infants. Iron deficiency, specifically IDA, was more strongly associated with neurodevelopmental delays in females than in males.

The results showed that the mean serum ferritin levels were significantly higher in female than in male infants, which is consistent with previous studies^9,17–20^. However, to date, little has been reported on the mechanisms underlying sex-related differences in ferritin levels during early childhood. This mechanism may be explained by hormone-mediated differences in metabolism. It is well known that serum insulin and leptin concentrations are different in male and female infants^21^. Sex differences in ferritin levels during infancy may also be associated with cord blood ferritin levels. A previous study showed a significantly lower concentration of serum ferritin in umbilical cord blood in males than in females^22,23^. Furthermore, the physiological characteristics in the first year of life, greater need for and use of iron due to accelerated growth and development, and progressive changes in the dietary supply and bioavailability of iron may result in variations in serum ferritin levels.

Studies on the association between serum ferritin and neurodevelopment performed decades ago showed that higher serum ferritin levels were associated with better neurodevelopmental function^24^. Similarly, sex differences were observed in the association between serum ferritin levels and neurodevelopment in our study. We found a linear relationship between serum ferritin levels and general quotient, gross motor, fine motor, language, and adaptive behavior scores in females but not in males. Recent research from Canada has found a stronger negative nonlinear relationship between serum ferritin and cognitive function in children aged 1–3 years, and recommends a serum ferritin level of 17 μg/L corresponding to the maximum level of cognition in children^25^. The pathophysiological pathways responsible for iron status and neurodevelopmental outcomes are complex and include dysfunctional myelination, neurotransmitter alterations, and endocrine pathways^26^. Iron deficiency is a common micronutrient deficiency primarily affecting children and women^27^. Iron is a key element in myelin production, neuron metabolism, and dopamine function. Iron deficiency during infancy can alter brain development, disrupt cognitive development, and exert long-term effects. Iron-mediated epigenetic mechanisms indicate that early-life iron deficiency directly causes stable changes in gene regulation across the lifespan, resulting in cognitive impairment and neuropsychiatric disorders^28^. Studies have shown significant differences in iron status between males and females. Girls, especially adolescents, have a high demand for iron to maintain their physical and psychological development^29^. To our knowledge, few studies have evaluated the relationship between serum ferritin and neurodevelopmental function in a sex-specific fashion, which may be associated with sex differences in hepcidin levels, which regulate neuronal ferroptosis in cognitive dysfunction^30^. It remains unclear how sex differences affect outcomes. Considering sex differences is important for developing preventive strategies for adverse neurodevelopmental effects due to iron deficiency.

This study has several limitations. A key limitation is the use of the database of a hospital information system. Consistent with similar studies, some data may have been incomplete or missing. Participants were included using non-random population-based sampling. The representativeness of the data may have been influenced by a selection bias. Although we carefully adjusted for potential confounding factors in our analyses, the database did not record some necessary confounders, such as iron supplementation and dietary habits; therefore, we did not adjust for them in our analysis.

## Conclusions

This study highlights the association between serum ferritin levels and neurodevelopment in infants aged 6–12 months with sex differences. Females with iron deficiency, especially those with IDA, are more susceptible to neurodevelopmental delays than males. Our study suggests that serum ferritin may have a sex-specific effect on neurodevelopment: females may have worse neurodevelopmental outcomes with iron deficiency and IDA. It may be necessary to consider the sex of infants when evaluating serum ferritin concentrations and providing recommendations for the nutrition of infants. Furthermore, there may be a need to develop sex-specific cutoff levels of ferritin in early childhood.

## Supplementary Information

Supplementary Information 1.

Supplementary Information 2.

## Data Availability

The datasets used and analysed during the current study are available from the corresponding author on reasonable request.

## Abbreviations

CI: Confidence intervals
CNBS-R2016: Children Neuropsychological and Behavior Scale-Revision 2016
IDA: Iron deficiency anemia
OR: Odds ratio
SD: Standard deviation

## References

[1] Wang W, Knovich MA, Coffman LG, Torti FM, Torti SV (2010). Serum ferritin: Past, present and future. Biochim. Biophys. Acta.

[2] McLean E, Cogswell M, Egli I, Wojdyla D, de Benoist B (2009). Worldwide prevalence of anaemia, WHO Vitamin and Mineral Nutrition Information System, 1993–2005. Public Health Nutr..

[3] Lozoff B, Jimenez E, Wolf AW (1991). Long-term developmental outcome of infants with iron deficiency. N. Engl. J. Med..

[4] Georgieff MK, Brunette KE, Tran PV (2015). Early life nutrition and neural plasticity. Dev. Psychopathol..

[5] Oatley H (2018). Screening for iron deficiency in early childhood using serum ferritin in the primary care setting. Pediatrics.

[6] Tran PV (2016). Prenatal choline supplementation diminishes early-life iron deficiency-induced reprogramming of molecular networks associated with behavioral abnormalities in the adult rat hippocampus. J. Nutr..

[7] Looker AC, Dallman PR, Carroll MD, Gunter EW, Johnson CL (1997). Prevalence of iron deficiency in the United States. JAMA.

[8] Rushton DH, Barth JH (2010). What is the evidence for gender differences in ferritin and haemoglobin?. Crit. Rev. Oncol. Hematol..

[9] Domellöf M (2002). Sex differences in iron status during infancy. Pediatrics.

[10] Wieringa FT (2007). Sex differences in prevalence of anaemia and iron deficiency in infancy in a large multi-country trial in South-East Asia. Br. J. Nutr..

[11] Ernawati F, Syauqy A, Arifin AY, Soekatri MYE, Sandjaja S (2021). Micronutrient deficiencies and stunting were associated with socioeconomic status in Indonesian children aged 6–59 months. Nutrients.

[12] Wang QP, Guo LY, Lu ZY, Gu JW (2020). Reference intervals established using indirect method for serum ferritin assayed on Abbott Architect i2000SR analyzer in Chinese adults. J. Clin. Lab. Anal..

[13] Guo Y, Deng YH, Ke HJ, Wu JL (2021). Iron status in relation to low-level lead exposure in a large population of children aged 0–5 years. Biol. Trace Elem. Res..

[14] World Health Organization. WHO guideline on use of ferritin concentrations to assess iron status in individuals and populations (WHO Guidelines Approved by the Guidelines Review Committee, 2020). 33909381

[15] Jin C-H (2016). Children Neuropsychological and Behavior Scale: Revision 2016.

[16] Li HH (2019). Comparison of the Children Neuropsychological and Behavior Scale and the Griffiths Mental Development Scales when assessing the development of children with autism. Psychol. Res. Behav. Manag..

[17] Sherriff A, Emond A, Hawkins N, Golding J (1999). Haemoglobin and ferritin concentrations in children aged 12 and 18 months. ALSPAC Children in Focus Study Team. Arch. Dis. Child..

[18] Emond AM, Hawkins N, Pennock C, Golding J (1996). Haemoglobin and ferritin concentrations in infants at 8 months of age. Arch. Dis. Child..

[19] Mireku MO (2016). Prenatal iron deficiency, neonatal ferritin, and infant cognitive function. Pediatrics.

[20] Pérez-Acosta A (2021). Cut-off points for serum ferritin to identify low iron stores during the first year of life in a cohort of Mexican infants. Matern. Child Nutr..

[21] Lönnerdal B, Havel PJ (2000). Serum leptin concentrations in infants: Effects of diet, sex, and adiposity. Am. J. Clin. Nutr..

[22] Tamura T, Hou J, Goldenberg RL, Johnston KE, Cliver SP (1999). Gender difference in cord serum ferritin concentrations. Biol. Neonate.

[23] Larsson SM (2019). When age really matters; ferritin reference intervals during infancy revisited. Scand. J. Clin. Lab. Invest..

[24] Lozoff B (2008). Dose-response relationships between iron deficiency with or without anemia and infant social-emotional behavior. J. Pediatr..

[25] Parkin PC (2020). Association between serum ferritin and cognitive function in early childhood. J. Pediatr..

[26] Bakoyiannis I (2015). An explanation of the pathophysiology of adverse neurodevelopmental outcomes in iron deficiency. Rev. Neurosci..

[27] Algarín C (2022). Cognitive control inhibition networks in adulthood are impaired by early iron deficiency in infancy. NeuroImage Clin..

[28] Barks AK, Liu SX, Georgieff MK, Hallstrom TC, Tran PV (2021). Early-life iron deficiency anemia programs the hippocampal epigenomic landscape. Nutrients.

[29] Li S, Zhao L, Yu D, Ren H (2022). Attention should be paid to adolescent girl anemia in China: Based on China Nutrition and Health Surveillance (2015–2017). Nutrients.

[30] Kong WN (2014). Sex differences in iron status and hepcidin expression in rats. Biol. Trace Elem. Res..

