# Supplementary figures and images for: Gender-specific association between serum ferritin and neurodevelopment in infants aged 6 to 12 months
Source: Sci Rep. 2023 Feb 13;13:2490. doi: 10.1038/s41598-023-29690-x (PMC9925425; doi:10.1038/s41598-023-29690-x)

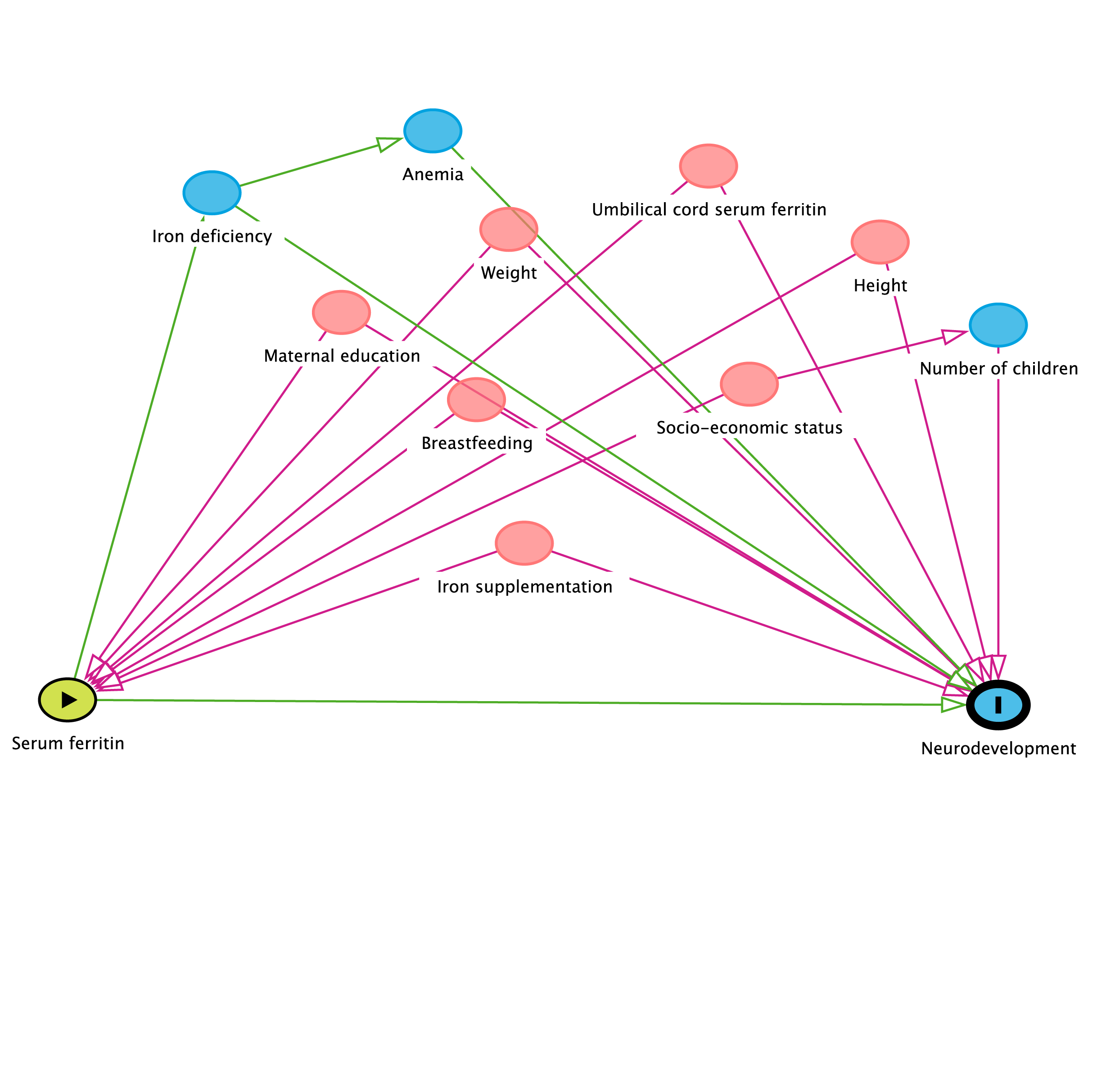

Supplement: Supplementary file 1 — Supplementary Information 1. [file 41598_2023_29690_MOESM1_ESM.tif]
