# Supplementary material for: Gender-specific association between serum ferritin and neurodevelopment in infants aged 6 to 12 months
Source: Sci Rep. 2023 Feb 13;13:2490. doi: 10.1038/s41598-023-29690-x (PMC9925425; doi:10.1038/s41598-023-29690-x)
Supplement: Supplementary file 2 — Supplementary Information 2. [file 41598_2023_29690_MOESM2_ESM.docx]

Table S1 Sex Differences in the associations between IDA and neurodevelopmental delays for males and females.

| Variable | Male | |  | Female | |  | *P*-Interaction |
| --- | --- | --- | --- | --- | --- | --- | --- |
|  | ORs (95% CI) | *P* value |  | ORs (95% CI) | *P* value |  | sex ∗ IDA |
| General quotient | 0.79(0.34, 1.89) | 0.606 |  | 4.88(1.74, 13.65) | 0.003 |  | 0.015 |
| Gross motor | 1.38(0.89, 2.13) | 0.155 |  | 1.93(0.95, 3.92) | 0.069 |  | 0.605 |
| Fine motor | 1.10(0.63, 1.93) | 0.730 |  | 2.58(1.13, 5.94) | 0.025 |  | 0.139 |
| Language | 1.12(0.64, 1.96) | 0.700 |  | 2.02(0.89, 4.58) | 0.092 |  | 0.242 |
| Personal-Social | 1.22(0.57, 2.59) | 0.611 |  | 2.96(1.01, 8.72) | 0.049 |  | 0.377 |
| Adaptive behavior | 0.93(0.50, 1.73) | 0.822 |  | 3.38(1.51, 7.57) | 0.003 |  | 0.030 |

Notes:

IDA: iron deficiency anemia (serum ferritin <12 ng/mL and hemoglobin <110 g/L).

The ORs were adjusted for maternal education, parity, feeding at six months, age of infant, height and weight.

*P* value for the interaction between sex and IDA, with the following variables also included in the model: maternal education, parity, feeding at six months, infant age, height and weight.
